# Documenting research in simulation science to enhance understanding for reusability

**DOI:** 10.1098/rsos.240776

**Published:** 2024-10-30

**Authors:** Sibylle Hermann, Jörg Fehr

**Affiliations:** ^1^ Institute of Engineering and Computational Mechanics, University of Stuttgart, Stuttgart 70569, Germany; ^2^ Cluster of Excellence SimTech, University of Stuttgart, Stuttgart 70569, Germany; ^3^ University Library, University of Stuttgart, Stuttgart 70569, Germany

**Keywords:** reusability, documentation, epistemology

## Abstract

One goal of Open Science is to promote reusability, which requires understanding and documentation. Reusability spans a spectrum from the straightforward reuse of existing materials to the extraction and adaptation of specific elements, depending on the maturity of the reused research and the research context. Beyond knowledge, understanding is crucial for enabling reusability. Simply reading an article is often insufficient; thus, publishing the underlying data and software is recommended. While the FAIR principles (Findable, Accessible, Interoperable, Reusable) facilitate the discovery and legal reuse of data and software through metadata, they fall short of promoting comprehensive understanding. By applying insights from the epistemology of simulation to computational mechanics research cases, we tested our hypothesis that the simulation process involves critical components beyond software and data that are essential to understand for reuse. Our findings indicate that reusability in these cases predominantly involves adjusting existing methodologies—a combination of different process steps from an epistemological perspective. Therefore, it is imperative to document not only learned knowledge but also the decisions regarding adjustments, assumptions and applications that concern the entire simulation process. Documentation is vital for understanding and herby enabling true reusability in scientific research, aligning with philosophical considerations of transparency and the nature of scientific knowledge.

## 1. Introduction

Have you ever tried to actually reuse research results from other researchers?

The concept of reusability has been discussed in numerous academic disciplines, with the realization that articles alone do not provide sufficient information to enable the reuse of the results presented (e.g. [1–3]). In the context of this article, the term ‘reusability’ is understood to include, but not be limited to, the related concepts of replicability and reproducibility, as discussed by Fehr *et al*. [4]: replicability is defined as the possibility to repeat the research with the same setting, resulting in the same outcome. The term ‘reproducibility’ refers to the ability to repeat the research by a different researcher in a different environment. In its broadest sense, encompassing all other terms, reusability is considered as the capacity to reproduce and replicate results in a different environment and context. The spectrum of reuse ranges from the reuse of the entire research project to the reuse of specific elements of the research, including methods, software or data. Reuse can be carried out in the same or in other research areas. Depending on the intended reuse of the results, a different depth of understanding is needed. Reuse can be viewed from the infrastructure side, in finding and providing access and the right to reuse the results, which is addressed by the FAIR principles [5] with the aspects of findability, accessibility, interoperability and reusability. From a scientific perspective, the requirements of the ‘Guidelines for safeguarding good research practice’ [6] take the research process and its reproduction into account: this includes publishing research data (guideline 13), quality assurance (guideline 7) and documentation (guideline 12), as well as research design (guideline 9), methods and standards (guideline 11). The guideline’s aim of reusability is to ensure the transparency of the research process. In summary, reusability encompasses several aspects, beginning with the scope and type of subsequent use, which requires different depth of understanding. Additionally, reusability depends on the possibility of retrieval and legal permission. In terms of content, reusability refers to the entire research process, which we will discuss in closer detail in this paper, examining simulation based on examples of computational mechanics.

In simulation, reusability is often associated with the publication of software and data. While software undoubtedly plays an essential role in research, simulation is not just about writing software. In fact, if that were the case, software engineers would be the main agents for simulation. Instead, many scientific disciplines seek to simulate phenomena related to their background, requiring a more comprehensive understanding of the research process beyond the software itself. Some scientific disciplines already address reusability for simulation. For example, Gunderson & Kjensmo [7, p.1645] define reusability in empirical AI research as ‘the ability of an independent research team to produce the same results using the same AI method based on the documentation made by the original research team’. Focusing on the medical field, Goodman *et al*. [8] suggest more specifically what should be reproduced: methods, results and inferential. Mullenendore *et al*. [3, p.3] analyse how and what needs to be done to achieve replicability for simulation models in ‘computational modelling’ in earth science. Mullendore *et al*. demand awareness of what to share: ‘Simply sharing model code doesn’t provide the level of understanding needed to easily build upon existing research’. This paper shows that problems about understanding and reproducing research concern all scientific disciplines in simulation science, but subject-specific peculiarities also must be addressed.

We focus our examination on computational mechanics in engineering sciences. Engineering science draws on principles from various branches of science, such as physics, chemistry and biology, and collaborates with experts from different scientific disciplines to achieve comprehensive solutions. Computational mechanics combines simulation and engineering science by applying mathematical and scientific principles to analyse and evaluate the performance of technical designs and systems. Simulation functions in this context as a means to an end, supplementing traditional experiments, often referred to as computer-based experiments, and occasionally also replacing them. In this context, Hey *et al*. [9] defined four paradigms for research evolution, from experiments over analytical theory to the third paradigm, numerical simulation. The fourth paradigm, proposed by Hey *et al*., is data-driven science, which is based on the idea that data itself can drive the discovery of new knowledge. Owing to the high complexity, that determines not only the content of the research, but also its execution in simulation, understanding in this article always refers to two levels: on the one hand, the content aspect of engineering research, and on the other hand, the increasingly complex science of simulation, which also requires a certain understanding of software development.

Looking deeper into the research process from an epistemological view reveals that, according to Gelfert [10], the process of addressing a scientific problem through computer modelling is commonly recognized as a multistep procedure. Decisions related to the problem take precedence before designing a simulation model. These decisions form an integral part of the research process, as they determine the choice and adjustments of software, method and model. Winsberg [11] argues that simulation is influenced by parameterization, numerical solution methods, mathematical tricks, approximations, idealizations, fictions, adjustments, function libraries, compilers, computer hardware, as well as trial and error. Lenhard & Hasse [12] goes even further in concluding that reusability is difficult to achieve because the transferability of the model is not given owing to parameterization, which depends on numerous factors, including the adjustment strategy used, the optimization method and the definition of boundary conditions. This epistemological view of simulation shows that, in addition to understanding the content of the research and the software, decisions are important. The simulation does not function as a pure representation of the physical world, and many adjustments are made that are not necessarily rationally comprehensible, but essential for a simulation to work. Reusability, therefore, also requires an understanding of the underlying decisions and adjustments.

However, the precise requirements of understanding for reuse remain unclear. From our perspective, the epistemology of simulation offers perspectives that can provide further insight that are not yet reflected in practice. In this article, we will (i) examine the characteristics of the simulation process of gaining knowledge from a software engineering and epistemological perspective, and (ii) how these specific characteristics apply to selected research cases in computational mechanics, all of which use one specific software, but do different kinds of research. Moreover, (iii) to gain more insight into the actual reuse in the research cases, an analysis was undertaken of the references cited in articles from the research cases.

Our findings indicate that from an epistemological perspective, research itself can occur at different points in the simulation process and happens mainly in the adjustment of models and methods. The computational mechanics research process in the selected research cases is built on so-called ‘SPH-Models’ (see §2.2), a methodological combination of the simulation process steps, complemented by the method. Whereby, the terminology defined based on epistemological considerations is used inconsistently. Furthermore, the ‘SPH-Models’ have different maturity levels, which influence the level of understanding needed for reuse.

In §2, we explain our approach and the methods used. In §3, we show how the transfer of the insights from the epistemology of simulation gives ideas about what is needed to achieve understanding for reusability and addresses these implications for future work in the discussion.

## 2. Methods

Our research assumes that not only research data and software are needed for reuse. The aim of our study was to gain an idea of what is needed to be documented to achieve understanding for reusability. To meet this aim, we answer the following questions:

- what characterizes the simulation research process of gaining knowledge?
- how does this characterization apply to the selected research cases? and
- how and what is reused in the selected research cases?

### 2.1. Collecting and analysing data

We have taken the following steps to answer these questions: to gain a comprehensive understanding of the characteristics of the simulation research process, we examined the simulation process from an epistemology and software engineering perspective by (i) reviewing the literature in this field, and (ii) comparing these findings with our selected research cases. The data collected included participant observation and semi-structured interviews [13], similar to a study in the same field by Horsch *et al*. [14]. Furthermore, articles and documentation of the chosen examples were evaluated. Besides, we have (iii) analysed the references in the articles of the selected research cases based on the following categories: to understand the context of the citation, we classified the citations into referring and reuse. In this context, "referring" is intended to indicate that a citation is included in a document to point to another article, even though the content of that article does not contribute to or support the current work. The term reuse refers to the use of data, concepts or theories previously established by others to enhance, sustain or build upon for their own work. It can also mean that the referenced article is used to set boundaries or differentiate the current work from that of the cited article. To gain a more profound understanding of these purposes of reuse of other research, we defined a further subdivision of the term reuse: adjustment, application, assumption and comparison. In this context, adjustment refers to an existing concept, combined with something new to refer to the broader idea, while application and assumption focus more on details. The term application is employed when the reference was used for an existing concept that was incorporated into the work itself in the form of formulas, models, theories or parameters. In contrast, assumption is used for refererences, when an existing concept was incorporated into the model without formulas. Comparison is used for an existing concept to compare or validate its results, methods or assumptions.

### 2.2. Case selection

We conducted a detailed investigation of research from research assistants and doctoral students in computational mechanics. Their common ground is the usage of the same research software, called Pasimodo [15]. Pasimodo was initially designed for the discrete element method (DEM), which is used to describe systems with independent solids interacting based on Newton/Euler equations. Pasimodo was then extended with a plugin for smoothed particle hydrodynamics (SPH), a mesh-free discretization method commonly applied in continuum mechanics [16,17] and well suited for systems with free surfaces or many changes in the state of aggregation. The research cases build on previous work from other researchers who have already completed their PhD. The study was conducted on the latest of roughly four generations of doctoral students, whose specific research is located in the following three areas.

#### 2.2.1. Laser beam welding

Laser beam welding is a joining technique that is becoming increasingly popular owing to its high welding speed, low thermal distortion and ease of automation. However, the process is susceptible to instability, which can result in the formation of defects such as pores and spatter. These can weaken the quality of the weld seam [18]. To gain an insight into the underlying physical mechanisms that cause these defects, numerical simulations are often used, given the limited accessibility of experimental observations. Modelling the laser beam welding process presents a significant challenge owing to the rapid changes in the interfacial properties between the solid metal, liquid melt and vapour phases. Therefore, in the selected research case of laser beam welding, the researcher’s focus was on simulating the physical phenomena that induce capillary instability and other disturbances of the process, such as pore formation [19] and spattering [20]. The goal of the simulation was to identify and reduce these phenomena systematically, building on work from predecessors. The research is based on a mature model that has been expanded to include the physics of the new phenomena of interest and was improved in terms of its methodology.

A total of five articles were analysed: two articles are concerned with methods [21,22], the other three with model adjustments [23–25].

#### 2.2.2. Deep-hole drilling

Deep-hole drilling is used to drill holes with a length-to-diameter ratio greater than 10 with applications in the automotive, aerospace, medical and food industry machinery sectors. This process includes various methods capable of drilling diameters less than one millimetre, all of which produce high-quality holes and achieve high productivity levels. However, deep-hole drilling is challenging owing to the high length-to-diameter ratio, particularly in single-lip drilling. The asymmetrical single-lip design results in non-vanishing forces, and evacuating produced chips is a major concern. Single lip drills have only one straight flute for chip evacuation. Consequently, cutting fluid flow and chip shape significantly influence process reliability, as long-shaped chips can cause jamming and potentially break the drill. Therefore, the main focus of the research in this research case was to develop a model to represent the jamming of the drilling chips during deep-hole drilling in a simulation, based on a model that already simulates the deep-hole drilling process but not the chip jamming. The model was built in close coordination with experimental investigations.

We analysed four articles, where two papers investigate fluid flow in connection with drilling geometry [26,27], one is an actual model of the drilling chip [28] and one investigates how to model the jamming [29].

#### 2.2.3. Particle damper

The growing importance of lightweight construction has led to a notable reduction in the weight of technical systems. However, this has also increased their vulnerability to unwanted vibrations. This vulnerability, when considered alongside the growing complexity of these systems, has prompted a re-evaluation of damping devices and the development of new methods to dissipate unwanted vibrational energy. Solid particle-filled dampers (PDs) have emerged as a popular alternative to conventional damping devices owing to their simple design and flexible ability to dissipate energy across a wide frequency range. In contrast to viscous dampers, PDs do not necessitate a fixed anchor point as an impulse source, thereby facilitating their installation on technical systems. The performance and influencing parameters of PDs have been subjected to extensive investigation through a multitude of experimental and numerical studies. These studies have identified several factors that impact PD performance, emphasizing the necessity for simulations to optimize particle dampers by quantifying properties such as frequency-dependent damping characteristics. Therefore, the aim of the researcher in this research case was to improve particle dampers specifically by minimizing dissipation within them. In the research case, simulation and experiment complemented each other, and simulation was used to better understand the phenomena inside the damper. The model consists of particles and fluid, where physical theories and design aspects are considered to form their material behaviour. The focus of the research lies on the behaviour of the particles and the influence of damping.

We analysed two publications, where one mainly contains experimental aspects [30] and the other simulation aspects, where mainly fluid and particle shapes are examined [31].

### 2.3. Validity analysis

For the analysis of our work, we proceeded inductively. The results are obtained for individual cases and then abstracted. So far, to our knowledge, no comprehensive study has been conducted to confirm or refute these results. The aim was not to enter into a methodological discussion in philosophical disciplines but to illuminate the research process in computational mechanics from various perspectives. In addition, the results of this research have been and will be presented at relevant conferences and discussed with experts of various disciplines. The terms model, theory and method are subject to varying interpretations owing to their diverse usage and definitions across scientific disciplines. This could result in different interpretations of some citations in our analysis. Each citation has been categorized according to its contextual placement within the text and the subject of the referenced article. While this method proved sufficient for our purposes, it could be argued that relying solely on sentence-level decisions is not sufficient for determining the extent of content reuse [32].

For the validity of the research findings, several measures are available. These measures include:

— discussions of results at internal and external meetings and conferences:

- internal discussions take place with individuals from the involved research group, providing an opportunity for critical review and feedback;
- presentations at the SimTech status seminar allow for external scrutiny and discussion of the research findings; and
- the Mathematical Research Data Initiative (MaRDI) [33] session at KLAIM [34] and the MaRDI Workshop meets information specialists [35] offered a platform for further evaluation and validation of the results.

— transfer of the method: the research findings can be transferred to other applications or scientific disciplines, serving as an additional check of the validity of the method.

## 3. Results

### 3.1. The process of gaining knowledge in simulation science

In this section, we first evaluate the research process from a software engineering perspective, referring mainly to Bungartz *et al*. [36], and compare it to the epistemological view on simulation. Winsberg’s [37] perspective serves as a crucial foundation for understanding the epistemology of simulation in our discussion, complemented by other philosophers from this area. These insights serve as a reference when we later examine their application in selected research cases.

Simulation is an ambiguous term that is defined both in computer engineering and in the epistemology of simulation. From a software engineering perspective, Bungartz *et al*. [36] understand simulation in a broader sense as an overall complex of pre-calculating or recreating a certain scenario and therefore as virtual experiments on a computer. Winsberg [11] expresses himself in a similar but more cautious way, in which he defines simulation in a broader sense as a method for studying systems comparable to experiments.^1^ According to Winsberg in this context, the simulation refers to a whole process that involves more than computing numbers, where the simulation process requires the creative use of various techniques and significant expertise to determine the reliability of the results [39]. Simulation in a narrower sense is defined by Bungartz *et al*. [36] as a calculation step in the process of reproducing a scenario that is already understood to gain a more in-depth insight or to predict scenarios that are not yet known. From an epistemological perspective, Winsberg [38] defines simulation in a narrower sense as a computer program that systematically examines how a mathematical model behaves by following a series of steps. Bungartz focuses more on the narrow sense of simulation, whereas Winsberg is more concerned with the broader sense. Both approaches describe the simulation as a process, which we will discuss in more detail below.

**Figure 1.**
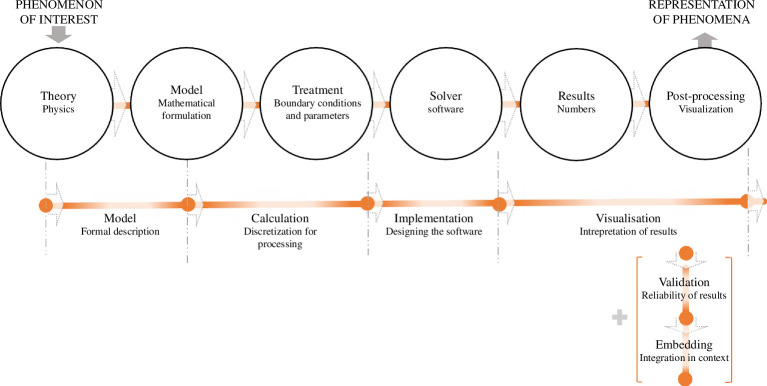
Comparing the simulation process from an epistemological and software engineering perspective (Hermann & Hengst [40]). The white circles represent the terminology and process steps from Winsberg [37] and are set in contrast with the approach of Bungartz *et al*. [36], represented through the orange lines.

Bungartz *et al*. [36] define the simulation process from a software engineering perspective as a pipeline (see figure 1): the first step is the model, which is defined as a simplified formal description of the object under consideration, forming the basis for the subsequent calculations. The next step of the pipeline is defined as calculation and refers to the process of preparing a model for processing on a computer to discretize the model in a way that allows it to be solved efficiently using the appropriate algorithms. The third step is the implementation, where the calculation of the algorithms on the target architecture(s) happens in designing the software to be scalable and following best practices. This is followed by the visualization, where the resulting data from a simulation run are interpreted. The last two steps are the validation to check the reliability of the results and the embedding where the simulation is integrated into a development or production process. To summarize, the process entails mapping a mathematical formulation and its discretization in software, as well as visualizing, validating and integrating the outcome into an underlying concept.

From an epistemological view [37], the phenomenon of interest raises the research question before the simulation process begins (see figure 1). This question is then addressed with the simulation process, resulting in a representation of phenomena. Theories are established physical or mechanical principles that serve as the foundation for the simulation.^2^ Based on the theory, a model is derived that translates the theory into mathematical formulas. The model is a mathematical representation of the theory. The model is discretized with the help of a solver that calculates the results step-by-step with treatments in the form of given parameters and initial conditions. The results, typically numbers, are processed through a post-processing step and presented in the form of a representation of phenomena^3^ usually depicted through figures. However, the numbers that come out of the solver are not the actual goal of the simulation process, but rather the representation of phenomena enriched by visualization, mathematical analyses and observations depicting relevant knowledge about the phenomenon of interest. Both approaches describe similar process steps, although the vocabulary is partly different and the content also differs. Bungartz *et al*. [36] begin the simulation process with the model, whereas Winsberg [37] first deals with the concept of theory, which forms the basis of the mathematical formulation of the model. Even if both approaches share a basic understanding, software engineering is more about mathematics and its discretization, while epistemology is much more concerned with the underlying creative process, which we will now look at in more detail.

**Figure 2.**
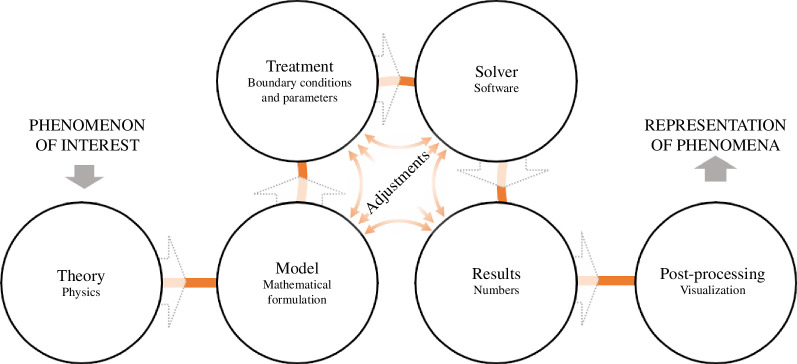
Simulation process as creative activity incorporating adjustments (Hermann & Hengst [41]). The simulation process involves adjustments based on computational limits and practical experience. Models must be simplified and enriched with features to correct approximation errors. These adjustments are made during the simulation process.

Winsberg [37] extends the software engineering view on the simulation process (see figure 1) incorporating creative activities in the form of adjustments (see figure 2). He argues that the actual simulation process must be ‘computationally contractible’ and does not fully correspond to reality, as models must be simplified to be solvable. This is done on the one hand by a simplified model but, much more frequently, by enriching the model with features that have nothing to do with the real world to correct errors caused by the approximation. These adjustments do not result from theory or physical intuition; they are much more determined by the limits of our ‘computational abilities’ [37]. Lenhard [43] holds a similar viewpoint to Winsberg [37], asserting that simulation models are not rationally constructed ‘clockworks’. He argues that the modularity of models is compromised through parameterization and ‘kluges’. Kluges, considered workarounds or quick fixes, are not typically considered good practice; nevertheless, they are a part of the simulation process. Lenhard [43] argues that there should be a balance in a model’s parameterization between adaptability and a well-grounded theoretical framework. Although these adjustments play such an important role, they are often not communicated. Lenhard [43, p.11] sees a ‘widespread reluctance against publishing about practices of adjusting parameters’, which stems in his opinion from ‘reservations against aspects that call for experience and art rather than theory and rigorous methodology’. However, particularly engineering science is highly dependent on experience and knowledge [44], where technological knowledge and the desired function play a crucial role. Furthermore, adjustments are made during the design process to achieve optimal handling [45] typically involving optimization [46]. So we even have two levels of adjustment: technical design and simulation. We can therefore state that although the simulation follows a process, it is subject to many adjustments (as shown in figure 2) where a lot of experience and knowledge is necessary to be able to evaluate the result and the process of the simulation.

Based on the software engineering and epistemological considerations, we divided the simulation process into the following six components: theory, model, treatment, solver, results and preprocessing. This classification can be approached differently.^4^ Terms in the simulation process can be interpreted disparately across scientific disciplines. Moreover, adjustments happen within the simulation process and in the technical design of the model to achieve an optimal solution. In light of the insights provided by the software engineering and philosophical discourse, the objective now is to examine how the scientists themselves approached the description of their research.

**Figure 3.**
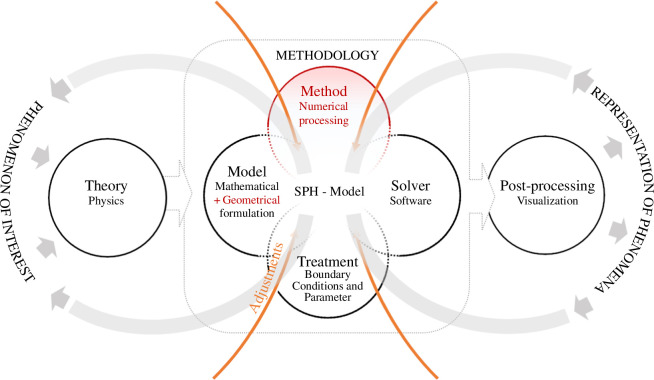
Simulation process derived from the research cases (Hermann & Hengst [42]). In the research cases, the process is represented less linearly and is based on the SPH-Model, which is a combination of the previously defined process steps model, treatment and solver, supplemented by the method. In addition to the mathematical formulation, the geometrical design of the objects also plays a role in the construction of the model.

In our analysis of articles from the selected research cases (table 1), we found significant variations of the identified terms in usage and interpretation. The terms theory and model are often used interchangeably, not just in the terminology of the authors but also in the reuse of other work. For example, the scattering effect in laser beam welding can be represented using either the Mie Theory or the Henyey–Greenstein model, both of which refer to a mathematical formulation. The model is not solely based on physical theories; it also incorporates geometrical considerations for technical design. For instance, the research case of deep-hole drilling largely involved geometric optimization of the drill and the drilling chips, where different modelling approaches were applied to find a suitable solution for simulating the effect of jamming. Despite not being explicitly mentioned in the description of the research process from the software engineering and epistemological perspective, the method is crucial in the examined cases. In this context, the term ‘method’ denotes a temporal and spatial discretization of the mathematical formulations of the model using the SPH approach. This is represented as calculation in Bungartz’s software engineering approach, yet it is not represented in the epistemological view of the process. In principle, the identified terms and their concepts are at least partially addressed in the research cases. However, this is not described as a research process; the focus is more on the methodology with a combination of model, treatment, solver and method, which is called the ‘SPH-Model’ (see figure 3).

**Table 1.** Components of the simulation process of the research cases based on the description of the researchers.

example	laser beam welding	deep-hole drilling	particle damping
theory	thermoelastic material behaviour (solid phase) and incompressible Newtonian fluid (liquid phase) with a transition region between the phases (mushy state)	Newton–Euler contact mechanics and incompressible Newtonian fluid	deformations, friction effects, hysteresis loop shocks and impulses, sound and energy radiation
model	solid and liquid phases as continuous media with underlying governing equations to describe the material behaviour. Enthalpy method for phase transitions: temperature is related to the enthalpy, which includes the latent heat of fusion in the relationship	drill and workpiece as solid state, coolant as fluid, different approaches for drilling chip (solid, plastic/elastic, linear elastic)	forces and movements of particles, kinetics of particle filling
treatment	Raytracer for the laser-material interaction, different thermal boundary conditions for the discretized workpiece (e.g. adiabatic or Dirichlet condition)	placing of chip, isotherm, free slip and Dirichlet boundary conditions	forced motion forces from momentum balances, how particles act on containers
solver	Raytracer and Pasimodo	Pasimodo and Neweul-M^2^	Pasimodo
methods	SPH: weakly compressible SPH, incompressible SPH, adaptive SPH	SPH, weakly compressible SPH, DEM, finite element method (FEM), floating frame of reference (FFR)	SPH, DEM

### 3.2. Implications of the ‘SPH-Model’

Based on the findings of the previous section, the implications of using the ‘SPH-Model’ are presented in more detail here.

The structure and maturity of the ‘SPH-Model’ differ in the three research cases (see table 2). The research takes place at different stages in the construction of the ‘SPH-Model’. In engineering, various levels of technology readiness (TRL) [47] are recognized. The technology readiness level expresses where the maturity of the research can be categorized. A high TRL indicates that research can be practically applied in industry. Transferred to the research cases, the laser beam simulation is the one with the highest maturity level. This applies both to the simulation of laser beam welding and also to the ‘SPH-Model’ considered here. There are a multitude of different approaches that can simulate laser beam welding, with numerous models in different research fields [48]. The ‘SPH-Model’, which underpins the research case, has been well-established with a large body of research incorporated into it. Thus, detailed phenomena can be represented through model expansion. In the context of deep-hole drilling, an existing model was in place. However, to answer the research question on how to simulate the jamming, the model requires adjustments to the technical design of the drill and drilling chips. This indicates a lower degree of maturity, in contrast to laser beam welding, where the emphasis was on optimization and physical theories. So far, the drilling process has been successfully executed without being able to represent the ‘new’ phenomenon of the drill chip’s jamming. The research from the studied case contributes to model building in this regard, although a complete solution has not yet been presented. The ‘SPH-Model’ of the particle damper is at the relatively basic conceptional level and is not yet fully developed and happens in close alignment with the experiment. All research cases, regardless of their maturity level, focus on the ‘SPH-Model’ and its adjustments.

**Table 2.** Description of the ‘SPH-Model’ and the conducted research.

example	laser beam welding	deep-hole drilling	particle damping
SPH-Model	the basic ‘SPH-Model’ has a high maturity level. In the ‘SPH-Model’, the discretized workpiece and heat sources, which are used to account for the energy input of the laser beam, are already included. These heat sources are treated as boundary conditions in the simulation and are computed by coupling the ‘SPH-Model’ with a ray tracer in a co-simulation	the ‘SPH-Model’ ranges at a medium maturity level; it is geometrically more complex than that for laser beam welding. It includes elements with different physical theories (fluid, drill, workpiece and drilling chips) and geometrical considerations (drill and drilling chips)	the ‘SPH-Model’ for the *particle damper* is still in its early stage of development
research	significant effort was put into optimizing the simulation performance by testing various forms of discretization within the SPH method. Another area of focus in the research was on understanding certain phenomena that couldn’t be fully grasped through experimental investigations. To this end, the existing ‘SPH-Model’ was enhanced with physical theories and their mathematical formulations	different chip geometries and modelling assumptions were explored to improve the simulation of jamming. This led to several hypotheses, including the need for adequate coolant flow for chip transportation and the impact of chip geometry on jamming. These hypotheses were then incorporated into the ‘SPH-Model’	the research involved both experiments and simulations to build the ‘SPH-Model’, which is in a less advanced stage compared to the laser and drilling models
adjustments	the phase transition is simplified in such a way that it is not simulated and, therefore, does not accurately represent the underlying physics. Once the system is definitively in a liquid state, the entire material is considered as liquid	the chip formation is simplified by adding chips from below into an empty drill hole, rather than simulating the actual process of material removal	friction is neglected in the dissipation mechanisms to reduce the number of degrees of freedom

In all research cases, adjustments are made to both the process and the ‘SPH-Model’, which cover more than the simulation process: it involves modifying the existing ‘SPH-Model’, to examine a particular phenomenon in detail, adapt the model geometrically if it is inadequate or further develop the model (see table 2). As such, the primary research aligns closely with figure 3, combining model, treatment, method and solver. The focus in the research cases lies on method optimization and model adjustment to better understand the phenomena under investigation. These adjustments materialize in the software, without explicit elucidation of the underlying assumptions and decisions. In the case of laser beam welding, the model is majorly constructed on physical theories. Adjustments are made to model new phenomena effectively within an already functioning simulation process. For example, to simulate the transition from solid to liquid (the so-called mushy phase), simplifications are incorporated to provide predictions regarding the occurrence of these phenomena. Moreover, substantial effort has been devoted to optimizing simulation performance by testing different discretization forms within the SPH method. In deep-hole drilling, adjustments are integral to the research. To understand how jamming can be modelled, various model approaches were assessed. Two challenges arise here: firstly, the interaction of several factors leading to jamming needs to be modelled. Secondly, these factors must be represented in a way that remains suitable for simulation. Furthermore, a co-simulation of multiple software products is necessary to map the diverse physical theories of solids and liquids. The research is based on an existing ‘SPH-Model’ without expecting an immediate functioning result. Therefore, the research outcome is not solely in the representation of phenomena but also serves as preliminary simulation experiments for future research. In particle simulation, adjustments are made to build a suitable ‘SPH-Model’, which is constantly compared with the experiment and aligned thereby with the adjustment process from our initial consideration. The adjustments also function to reduce complexity, which also includes an intentional non-physicality that allows, for example, the assumption of material properties of the particles that do not exist in reality. Based on these results, conclusions can then be drawn about optimal material compositions for damping. The ‘SPH-Models’ are based on the work of predecessors, where the ‘SPH-Model’ serves as an existing representation of phenomena, enhanced by additional adjustments that previous ‘SPH-Models’ could not address. Therefore, the research necessitates a combination of specialized knowledge domains, including expertise in fundamental physics and technical construction, proficiency in software development and an in-depth grasp of the methodological approach of SPH. Furthermore, it is essential to assess the current state of the ‘SPH-Model’ to accurately estimate the depth of knowledge that must be acquired to successfully answer the research question.

**Figure 4.**
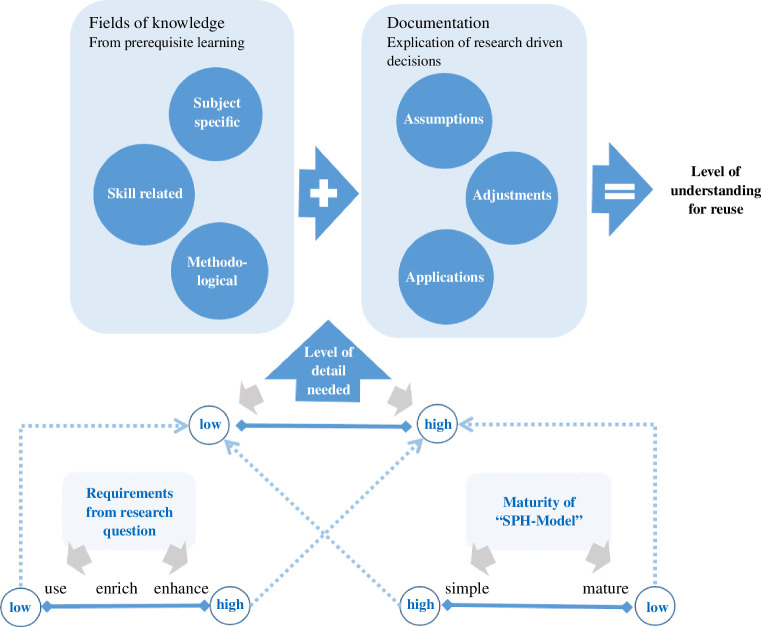
Depth of understanding for reusability (Hermann & Hengst [49]). The graphic represents the relationship between knowledge, documentation and the maturity level of a model for reusing research results. The arrows indicate that a higher maturity level of the model reduces the need for detailed understanding, whereas a lower maturity level necessitates a more precise understanding of the model’s parts. Additionally, documentation of assumptions, adjustments and decisions made during research is crucial for the reuse of results.

From the findings described above, we deduce that both knowledge and documentation are required (see figure 4) to be able to identify what we need to understand for reusing the research results. The required knowledge and documentation depend on the maturity level of the model and the research question. Based on the maturity of the model, a more detailed understanding of the parts of the ‘SPH-Model’ is required. A lower maturity level implies a more precise detailed understanding, in contrast to a fully developed model in which a less detailed understanding of the ‘SPH-Model’ is required. The knowledge, which is usually acquired through a university degree, we divide into the following areas: subject-specific (engineering), skill (programming) and methodological (SPH) knowledge. However, for the results to be reusable, it is essential to have an understanding of the underlying decisions related to adjustments, assumptions and applications, which are known only to the researcher conducting the simulation. This type of knowledge cannot be considered to be acquired through learning. Rather, it is obtained through the process of research. It is imperative that this accumulated body of knowledge, created by those who preceded the research, is documented. Therefore, it is essential to document the decisions made regarding adjustments, assumptions and applications to enable reuse of the results.

We have now worked out that understanding for reuse involves a combination of knowledge and documentation. Furthermore, the necessary depth of understanding depends on the maturity of the model to be reused and the research question. In the next step, we will analyse how the reuse in the research cases happened, to gain a more profound understanding of what needs to be documented for reusability.

### 3.3. Reuse within the research cases

We conducted a thorough examination of the citations in all published articles from the research cases, where the researchers were the main authors, to gain a more profound understanding of the knowledge, the researchers built upon to address their research questions. Therefore, we clustered and analysed the sentences, in which references were included, with the terms from §3.1: theory, model, treatment, solver complemented with method and ‘SPH-Model’ and analysed in the first step if the citation was used for reference (for citations, which are not further used) and reuse. Since reuse can be interpreted in different ways and contexts, we further differentiated into adjustment, application, assumption and comparison (see §2 for definitions).

**Figure 5.**
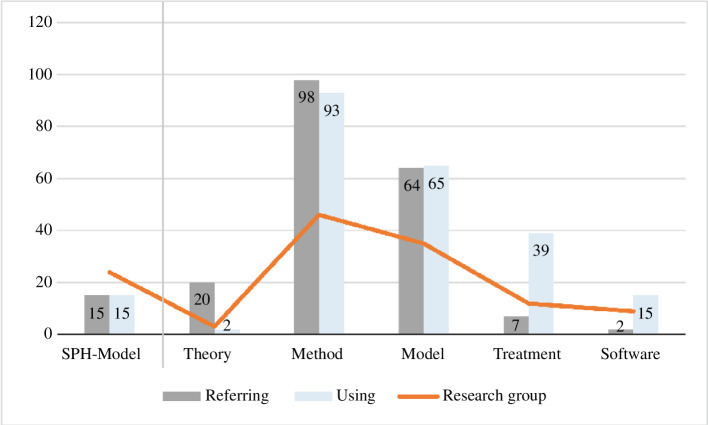
Number of references of publications in total and within the own research group. All published articles (11) from the three research cases with a total of 441 references were evaluated [32].

The citations predominantly reference external work for models and methods, while focusing on modifications to the ‘SPH-Model’. The citations often refer to previous work on the ‘SPH-Model’, but the authors do not delve into the details of the work of their predecessors. Instead, they focus on the changes they have made themselves. When adapting the methods and models, they mainly cite external work. Theories are generally cited as references, while treatments and solver are mainly cited for reuse. Both models and methods are cited for reference and reuse, as shown in figure 5. The reuse is subject to different purposes: the citations are used primarily for applications of the model, method and treatment, while adjustments are more related to the model. The assessed citations are primarily used for applications. These citations, which incorporate existing concepts, are chiefly applied to the method, but they also relate to the model, solver and treatment. Adjustments, on the other hand, are only related to the model and method. Assumptions were made across all categories. Citations for comparison were made for the model, method and treatment, but not for the solver. In summary, the process of reusing involves adapting the ‘SPH-Model’ to suit the research question. This is done by making assumptions, adjustments and applying it in relevant contexts. Furthermore, other sources are used for comparisons.

The objective of this section was to examine the ways in which the researcher reused knowledge through an analysis of their citations. The findings indicate that it is difficult to gain a comprehensive understanding, without reviewing all the referenced articles, even though the underlying decisions are partly implicitly available. The ‘SPH-Model’, which was primarily reused and modified in accordance with the specific research question, was accessible to the researchers. Furthermore, the researchers had access to the underlying software and, in the majority of cases, to the predecessor who could provide additional insights. Otherwise, it would not be feasible to reuse the ‘SPH-Model’ based solely on the articles. While articles present the research, it is not their intended purpose to document every decision. Therefore, we propose to give a short overview of the decisions that underlie the research. We put forth a proposed solution in the form of a ‘reuse table’, which illustrates the reuse, adaptation and modification of the components software, model, method and treatment. To illustrate this proposed solution, we have provided an outline of such a ‘reuse table’ using laser beam welding as an example, which has been developed based on our analyses and discussions with the researcher (see table 3).

**Table 3.** Example for reuse table of laser beam welding.

component	reused	adjusted	new
software	Pasimodo	implementation in the software of adjustments of model, method and treatment	defined SPH as a plugin that is called by the core
model	predecessor PhD Zerbe [18]	the transition is not simulated: as soon as everything is certainly liquid, everything is considered as liquid, before solid	improved surface finish model
method	SPH	gas phase with Rieman SPH	SSPH
treatment	predecessor PhD Zerbe [18]	different material and process parameters	dispersion

## 4. Discussion

Our study indicates that understanding the research process is crucial for reusing simulation results. The primary finding is that reuse needs understanding in the form of learned knowledge and documentation. The necessary depth of understanding depends on the research question and the maturity of the reused ‘SPH-Model’, which includes the methodological combination of model, treatment, solver and method. Adjustments, often undocumented, play a significant role in the simulation process and the ‘SPH-Model’.

Our study aligns with Winsberg’s [37] and Lenhard’s [43] assertions that simulation models are not purely theoretical constructs but involve creative adjustments and practical experience. The current focus of discussion within the research data community does not address this issue [5]. The emphasis is on the dissemination of data and software through publication, which, while also necessary, is, according to our results, not sufficient. We showed that in the investigation of the use of specific research software, very different phenomena and aspects of research are addressed. The results in numbers and software do not include documentation of the decisions behind the research results, even if there is already some preliminary work in other specialist areas on the documentation of models [50], software documentation [51] and documentation for reusability for a specific research field [52].

These best practices are important but are still far from being established in science. Moreover, the discussion about the documentation is often about how rather than what [51]. New approaches are based on automation [53], making it possible to gain a more profound understanding of the research work or use machine learning to help with documentation [54,55]. These approaches are certainly helpful, but it should still be clear what exactly should be documented, to achieve reusability. However, as long as scientific evaluation does not reward documentation, there is little incentive to invest the considerable effort that documentation entails.

Another concern involves the necessary level of detail in documentation, as there is uncertainty about how much information is required. This presents the challenge of implicit knowledge: researchers often do not realize the extent of the knowledge they have that needs to be shared. Gorman [56] argues that implicit knowledge is an important part of technology transfer and cannot be unconditionally transferred with documentation. Similarly, Lenhard & Hasse [12] highlight that the transferability required for reuse is challenging to attain, given that the models are more complex than they initially appear, in particular owing to the parametrization. As a possible solution, we suggest more explicit documentation of what has been reused and what kind of adjustments have been made in the individual steps. The first step is to consider what tacit knowledge is available, as experts usually are not aware of this themselves, as Gorman [56] points out. Consciously completing a spreadsheet or questionnaire can help to convey this implicit knowledge. Additionally, projects such as MaRDI are already developing ontologies for mathematical models and algorithms [57]. This could serve as a precedent for acknowledging, similarly to research data and software, the models and algorithms used in simulations, eliminating the need to detail them in every article. Some research areas try to establish standards, like TRAnsparent and Comprehensive model Evaluation (TRACE), where this standard is only useful if everyone is involved in using it [58]. Transferability to other research fields is certainly possible. Nevertheless, this standard must first be introduced and then taught. Therefore, as a first step, we advocate for a more pragmatic approach, as outlined in the table presented in the results (table 3), which can certainly be expanded upon in the future. Another idea is ‘epistemic metadata’, which is discussed in Horsch *et al*. [14]: the authors introduce the idea of providing information in the metadata about what the authors claim to know, how others should accept the results as knowledge and information about validation, verification and reusability of the research. This approach aligns with our results and, similar to the TRACE standard, can serve as a foundation for further research.

Traditional engineering education often focuses on application and optimization. However, with the rise of Open Science, there’s a growing need to orient engineers more towards scientific methods [59]. In the research software discussion, the focus is often on improving the training of engineers in software development. This is important and contributes to the necessary knowledge part of our explanation of reuse (figure 4). However, the meta-knowledge that the simulation method brings should also be part of the training, as should knowledge about the correct documentation.

This is precisely where we see the strength of our study, in which we bring the epistemological discussions into practical examples from computational mechanics and thus provide new impetus. The limitations of our study are the primary focus on specific research cases from one research group, which may not fully capture the nuances of simulation processes in engineering and other scientific disciplines. To enhance the generalizability of our findings, future research should include a more diverse range of cases. Additionally, we mainly refer to two sources to examine the simulation process from software engineering and epistemology perspectives. This limited scope does not address the validation and embedding of results as proposed by Bungartz *et al*. [36], which is also relevant to the epistemological discussion. Bringing in more perspectives and incorporating a broader literature review was beyond the scope of this study, but it would provide a more comprehensive understanding of the simulation process. Unexpectedly, we found that ‘SPH-Models’ as a methodological approach, rather than the simulation process itself, were influential in our selected research cases, where the main kind of reuse occurs through direct handover to successors. Nevertheless, the transfer of knowledge and internal documentation is often perceived as inadequate in these cases as well [51]. Whether these findings are transferable to other areas of research remains to be seen, and future studies should investigate if other research cases have similar methodological approaches that are reused.

The Deutsche Forschungsgemeinschaft’s emphasis on Open Science [60] highlights the growing importance of thorough documentation, extending beyond the article itself to include the methods and the steps that led to the research results. This involves documenting not only the research process but also the decisive factors that shaped the research method’s design. We have demonstrated that the research procedure in computational mechanics encompasses a broader scope than just research software. It is essential to delve more deeply into discussing the research design, methodologies employed and rationale behind selecting a specific method. By drawing insights from the epistemology of simulation, valuable lessons can be adapted to computational mechanics. Our next objective is to integrate these insights more tangibly into the documentation. Furthermore, we aim to enhance the integration of documentation within the realms of Open Science and research evaluation. We think that a pragmatic solution for documentation will lead to increased and varied reuse.

## Data Availability

All data are available at [32].

## Footnotes

^1^
In the philosophical debate surrounding whether simulation can be regarded as an experiment or an independent methodology, there is currently no consensus [38]. However, this is not relevant to our discussion here, as the discussion is being conducted at a higher level of abstraction.

^2^
Winsberg [11] argues that simulation is ‘downwards’ because it starts with an established theory to examine specific phenomena and does not begin with an observation that leads to a theory, as is common in traditional philosophy of science discussions.

^3^
Winsberg refers to this as a model of phenomena. However, to avoid confusion owing to the repeated use of the term ‘model’ in both the process and the phenomena, we have chosen to use the term ‘representation of phenomena’.

^4^
For instance, theory and model, treatment and solver, as well as results and post-processing are often grouped together.
